# Prevalence and Clinical Significance of Up-Sloping ST-Segment Depression in Patients With Non-ST-Segment Elevation Myocardial Infarction

**DOI:** 10.14740/cr422w

**Published:** 2015-10-25

**Authors:** Naoki Misumida, Akihiro Kobayashi, Paul Schweitzer, Yumiko Kanei

**Affiliations:** a Department of Internal Medicine, Mount Sinai Beth Israel Hospital, New York, USA; b Department of Cardiology, Mount Sinai Beth Israel Hospital, New York, USA

**Keywords:** Non-ST-segment elevation myocardial infarction, Up-sloping ST-segment depression, Electrocardiogram

## Abstract

**Background:**

Up-sloping ST-segment depression has not been historically considered as representing ischemia as this electrocardiographic change can be seen in normal subjects during exercise stress testing or tachycardia. We aimed to clarify the prevalence and clinical significance of up-sloping ST-segment depression in patients with non-ST-segment elevation myocardial infarction (NSTEMI).

**Methods:**

We performed a retrospective analysis of 330 consecutive patients with NSTEMI who underwent coronary angiography. ST-segment depression ≥ 0.05 mV in more than two contiguous leads was recorded and categorized as being up-sloping or non-up-sloping.

**Results:**

Of 330 patients, 109 patients (33%) had ST-segment depression; six of these patients had up-sloping ST-segment depression. All six patients with up-sloping ST-segment depression had a culprit lesion and underwent in-hospital revascularization. Three of these six patients had a culprit lesion in the left anterior descending artery; the culprit lesion in two others was in the left circumflex artery, while one patient had severe three-vessel disease. No statistically significant difference was found in the rate of in-hospital revascularization between patients with up-sloping and non-up-sloping ST-segment depression (100% vs. 75%, P = 0.33).

**Conclusions:**

Patients with up-sloping ST-segment depression had a comparable rate of in-hospital revascularization compared to those with non-up-sloping ST-segment depression, suggesting that up-sloping ST-segment depression should be recognized as a manifestation of ischemia in NSTEMI.

## Introduction

The current guideline for the management of non-ST-segment elevation acute coronary syndrome includes new or presumably new ST-segment depression as one of the indications for early invasive strategy within 24 h [1]. Among several types of ST-segment depression, up-sloping ST-segment depression has not been historically considered as representing ischemia as this electrocardiographic change can be seen in normal subjects during exercise stress testing or tachycardia [2-4]. Indeed, the current guideline for myocardial infarction includes only horizontal and down-sloping ST-segment depression as a manifestation of ischemia [5]. However, up-sloping ST-segment depression has been increasingly recognized as a sign of ischemia, and up-sloping ST-segment depression with a tall positive T waves in precordial leads was observed in 2% cases of anterior myocardial infarction [6]. The need for guideline revision to include up-sloping ST-segment depression as a possible manifestation of ischemia has been proposed [7]. Nevertheless, the prevalence and clinical significance of up-sloping ST-segment depression in non-ST-segment elevation myocardial infarction (NSTEMI) have not been fully clarified. We aimed to clarify the prevalence and clinical significance of up-sloping ST-segment depression in patients with NSTEMI.

## Methods

We retrospectively reviewed all patients who underwent coronary angiography between January 2013 and June 2014 at our institution. Inclusion criteria were: 1) a troponin level greater than the 99th percentile reference value before cardiac catheterization; 2) chest pain (or anginal equivalent) or ischemic change on the electrocardiogram including ST-segment depression (≥ 0.05 mV) or T-wave inversion (≥ 0.1 mV) in two or more contiguous leads; and 3) absence of ST-segment elevation or new left bundle branch block on the electrocardiogram. Exclusion criteria were: 1) cardiac catheterization more than 5 days after presentation; 2) bundle branch block, electrocardiographic left ventricular hypertrophy (LVH), or ventricular paced rhythm; 3) other identifiable causes of troponin elevation; and 4) insufficient data for analysis.

The present study complied with the Declaration of Helsinki and was approved by the institutional review board. Patients’ demographic data, risk factors and admission characteristics were obtained. Cardiac troponin I levels were measured using a second-generation VITROS^®^ Troponin I assay (Ortho-Clinical Diagnostics Inc., NJ, USA). The upper limit of normal for troponin I was 0.034 μg/L. Electrocardiograms obtained on presentation were reviewed by two independent reviewers. ST-segment shifts were measured at the J point for ST-segment elevation and depression. ST-segment depression ≥ 0.05 mV in more than two contiguous leads was recorded and categorized as being up-sloping or non-up-sloping (either horizontal or down-sloping) based on the ST-segment morphology in the lead with the most prominent ST-segment depression. Left ventricular ejection fraction was assessed by transthoracic echocardiography using either the Teichholz equation or the biplane Simpson’s method.

All patients underwent cardiac catheterization within 5 days of presentation. An independent cardiologist blinded to the clinical data reviewed all coronary angiography results. Obstructive coronary artery disease (CAD) was defined as stenosis greater than or equal to 70% (50% in the left main coronary artery). Angiographic findings including the number of diseased vessels, antegrade coronary flow according to the thrombolysis in myocardial infarction (TIMI) criteria, and pre-procedural thrombus grade according to TIMI study group were recorded. The infarct-related artery was determined based on the findings shown on electrocardiography, echocardiography, and coronary angiography. The primary outcome was in-hospital revascularization. In addition, in-hospital mortality, presence of heart failure and cardiogenic shock, and length of hospital stay were recorded.

Data are expressed as the number (percentage) or median (interquartile range). Continuous variables were compared using the Wilcoxon rank sum test. Dichotomous variables were compared using the Chi-squared test or Fisher’s exact test, as appropriate. Two-sided P-values < 0.05 were considered statistically significant. All statistical analyses were performed with R software (version 3.0.1).

## Results

Of 330 patients included in the final analysis, 109 (33%) patients had ST-segment depression. Patients with ST-segment depression had a higher rate of in-hospital revascularization than did those without ST-segment depression (76% vs. 63%, P = 0.02).

Baseline characteristics and angiographic findings of patients with up-sloping and non-up-sloping ST-segment depression are summarized in Table 1. Of the 109 patients with ST-segment depression, six patients had up-sloping ST-segment depression. Patients with up-sloping ST-segment depression had a higher peak troponin value and lower left ventricular ejection fraction than those with non-up-sloping ST-segment depression. All six patients with up-sloping ST-segment depression had a culprit lesion and underwent in-hospital revascularization. In-hospital mortality was higher in patients with up-sloping ST-segment depression than in those with non-up-sloping ST-segment depression, and two patients with up-sloping ST-segment depression died due to cardiogenic shock and sudden death, respectively.

**Table 1:** Baseline Characteristics, Laboratory Data, Angiographic Findings and Clinical Outcomes

	Up-sloping ST depression (n = 6)	Non-up-sloping ST depression (n = 103)	P value
Baseline characteristics and risk factors			
Age (years)	59 (49 - 69)	67 (60 - 78)	0.24
Men (%)	5 (83)	63 (61)	0.41
Hypertension (%)	4 (67)	80 (78)	0.62
Diabetes (%)	1 (17)	38 (37)	0.42
Hyperlipidemia (%)	2 (33)	62 (60)	0.23
Previous MI (%)	1 (17)	16 (16)	1
Previous PCI (%)	2 (33)	30 (29)	1
TIMI risk score 5 - 7	2 (33)	46 (45)	0.69
Hemodynamic, laboratory data and echocardiogram findings			
Systolic blood pressure (mm Hg)	120 (114 - 132)	144 (125 - 161)	0.04
Heart rate (beats/min)	90 (78 - 106)	81 (69 - 95)	0.51
Killip class > 1 on admission (%)	1 (17)	12 (12)	0.54
Peak troponin I (µg/L)	73.2 (15.6 - 253)	3.04 (0.27 - 11.0)	0.006
Left ventricular ejection fraction (%)	43 (39 - 49)	60 (45 - 62)	0.04
Angiographic findings and in-hospital events			
Interval^a^ (days)	0.4 (0.3 - 0.8)	1.0 (0.3 - 2.1)	0.22
Obstructive CAD (%)	6 (100)	93 (90)	1
Multi-vessel disease (%)	4 (67)	71 (69)	1
Left main/three-vessel disease (%)	2 (33)	43 (42)	1
Pre-procedural TIMI grade 0 - 1 flow (%)	3 (50)	32 (31)	0.38
Angiographic thrombus (%)	6 (100)	48 (47)	0.01
High-grade thrombus (TIMI grade 4 - 5) (%)	4 (67)	24 (23)	0.04
In-hospital revascularization (%)	6 (100)	77 (75)	0.33
In-hospital PCI (%)	5 (83)	60 (58)	0.4
In-hospital CABG (%)	1 (17)	17 (17)	1
In-hospital outcomes			
In-hospital all-cause death (%)	2 (33)	0 (0)	0.003
In-hospital heart failure (%)	1 (17)	15 (15)	1
In-hospital cardiogenic shock (%)	1 (17)	3 (3)	0.22
Length of stay (days)	7.6 (5.2 - 13.5)	5.8 (3.0 - 9.4)	0.22

^a^Interval from presentation to catheterization. Data are expressed as number (percentage) or median (interquartile range). CAD: coronary artery disease; MI: myocardial infarction; PCI: percutaneous coronary intervention; TIMI: thrombolysis in myocardial infarction; CABG: coronary artery bypass grafting.

**Figure 1:**
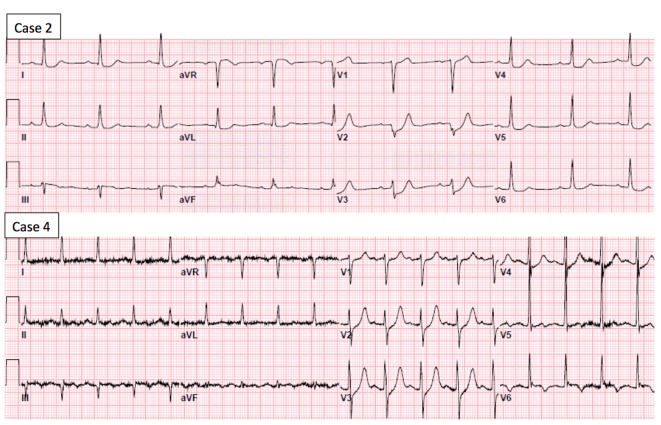
Electrocardiogram of case 2 shows up-sloping ST-segment depression in leads V2 and V3, and horizontal ST-depression in leads V4 to V6, I and aVL. Electrocardiogram of case 4 shows up-sloping ST-segment depression in leads V2 and V4.

Clinical data, electrocardiographic and angiographic findings, and in-hospital outcomes of the six patients with up-sloping ST-segment depression are summarized in Table 2. The electrocardiograms obtained in case 2 and case 4 are shown in Figure 1. Three of the six patients had a culprit lesion in the left anterior descending (LAD) artery and two of the three remaining patients had a culprit lesion in the left circumflex artery. One patient had severe three-vessel disease and underwent urgent coronary artery bypass grafting.

**Table 2:** Clinical Data, Electrocardiographic and Angiographic Findings, and In-Hospital Outcome of the Six Patients With Up-Sloping ST Depression

Case	Age	Sex	Symptom duration	Leads with ST depression	Interval^a^	IRA	TIMI flow	Collateral grade	No. of vessels	Procedure	Outcome
1	66	M	45 min	V3-V6	9 h	LAD	2	0	1 VD	PCI	-
2	70	M	90 min	V2-V6, I, aVL	7 h	LAD	0	2	2 VD	PCI	-
3	47	M	1 day	V4-V6, I, II, aVF	34 h	LAD	3	0	1 VD	PCI	-
4	91	M	12 h	V2-V4	9 h	LCX	0	0	3 VD	PCI	Death
5	48	F	2 days^b^	V2-V6, I, aVL	4 h	LCX	0	1	2 VD	PCI	Death
6	52	M	2 days^b^	V4-V5	23 h	3 VD^c^	2	0	3 VD	CABG	-

^a^Interval from presentation to catheterization. ^b^Intermittent crescendo chest pain. ^c^Severe stenosis (> 95%) in all three major coronary arteries. IRA: infarct-related artery; TIMI: thrombolysis in myocardial infarction; PCI: percutaneous coronary intervention; CABG: coronary artery bypass grafting; LAD: left anterior descending; LCX: left circumflex.

## Discussion

The current guideline for myocardial infarction does not include up-sloping ST-segment depression as a manifestation of ischemia [5]. Upsloping ST-segment depression in leads V1 to V6 with a positive symmetrical T wave was observed in about 2% of the patients with anterior myocardial infarction [6, 8]. In the present study, similar electrocardiographic findings were present in two patients with a culprit lesion in proximal LAD artery (cases 1 and 2). The remaining one patient with a culprit lesion in the proximal LAD artery (case 3) presented 1 day after symptom onset and had up-sloping ST-segment depression without a tall T wave. Considering its association with high-risk angiographic features such as proximal lesion and high rate of impaired coronary flow, up-sloping ST-segment depression in leads V1 to V6 with a positive symmetrical T wave has been proposed to be a “STEMI equivalent” that requires immediate coronary angiography [8, 9]. In the present study, the two patients with this electrocardiographic finding did not undergo immediate coronary angiography, highlighting a limited awareness of this high-risk nature of this finding.

Another possible cause of anterior ST-segment depression is posterior infarction. The presentation of posterior STEMI with ST-segment depression in leads V1 to V4 is well established finding [10]. In the present study, two patients with up-sloping ST-segment depression (cases 4 and 5) had a culprit lesion in the dominant left circumflex artery, consistent with posterior infarction. Upsloping ST-segment depression in precordial leads has been described in a patient with left circumflex artery occlusion [11]. It seems that up-sloping morphology of ST-segment depression occurred as a result of a mirror image of terminal T-wave inversion. ST-segment depression with positive T waves in leads V2 and V3 may represent an advanced electrocardiographic stage of posterior myocardial infarction [12]. This theory is consistent with the late presentation of the patients with left circumflex artery occlusion in the present study (cases 4 and 5).

Another possible cause of anterior ST-segment depression is multi-territory ischemia. One patient (case 6) had critical stenosis in all three major coronary arteries. Up-sloping ST-segment depression secondary to severe multi-vessel disease has been reported [13]. Our study suggested that it would be prudent to recognize up-sloping ST-segment depression as a manifestation of ischemia in the setting of NSTEMI. However, it may be difficult to distinguish up-sloping ST-segment depression due to ischemia from tachycardia-induced up-sloping ST-segment depression as tachycardia itself can cause up-sloping ST-segment depression and troponin elevation due to demand ischemia [4, 14].

The present study has several limitations. Firstly, analysis is subject to the usual constraints associated with a retrospective observational study. Second, our study cohort only included patients with NSTEMI who underwent coronary angiography, excluding patients with up-sloping ST-segment depression without troponin elevation or coronary angiography and limiting generalizability of the results.

### Conclusions

Our study demonstrated that patients with up-sloping ST-segment depression in the setting of NSTEMI had a comparable rate of in-hospital revascularization compared to those with non-up-sloping ST-segment depression, suggesting that up-sloping ST-segment depression should be recognized as a manifestation of ischemia in the setting of NSTEMI.
